# Exploring the Causal Effect of Constipation on Parkinson’s Disease Through Mediation Analysis of Microbial Data

**DOI:** 10.3389/fcimb.2022.871710

**Published:** 2022-05-11

**Authors:** Shih-Chen Fu, Ling-Chieh Shih, Pei-Hua Wu, Yi-Chen Hsieh, Chung-Han Lee, Sheng-Hsuan Lin, Hsiuying Wang

**Affiliations:** ^1^ Institute of Statistics, National Yang Ming Chiao Tung University, Hsinchu, Taiwan; ^2^ Department of Medicine, National Yang Ming Chiao Tung University, Taipei, Taiwan

**Keywords:** Parkinson’s disease, constipation, microbiome, intestinal microbial changes, mediation analysis

## Abstract

**Background and Aims:**

Parkinson’s disease (PD) is a worldwide neurodegenerative disease with an increasing global burden, while constipation is an important risk factor for PD. The gastrointestinal tract had been proposed as the origin of PD in Braak’s gut–brain axis hypothesis, and there is increasing evidence indicating that intestinal microbial alteration has a role in the pathogenesis of PD. In this study, we aim to investigate the role of intestinal microbial alteration in the mechanism of constipation-related PD.

**Methods:**

We adapted our data from Hill‐Burns et al., in which 324 participants were enrolled in the study. The 16S rRNA gene sequence data were processed, aligned, and categorized using DADA2. Mediation analysis was used to test and quantify the extent by which the intestinal microbial alteration explains the causal effect of constipation on PD incidence.

**Results:**

We found 18 bacterial genera and 7 species significantly different between groups of constipated and non-constipated subjects. Among these bacteria, nine genera and four species had a significant mediation effect between constipation and PD. All of them were short-chain fatty acid (SCFA)-producing bacteria that were substantially related to PD. Results from the mediation analysis showed that up to 76.56% of the effect of constipation on PD was mediated through intestinal microbial alteration.

**Conclusion:**

Our findings support that gut dysbiosis plays a critical role in the pathogenesis of constipation-related PD, mostly through the decreasing of SCFA-producing bacteria, indicating that probiotics with SCFA-producing bacteria may be promising in the prevention and treatment of constipation-related PD.

**Limitations:**

1) Several potential confounders that should be adjusted were not provided in the original dataset. 2) Our study was conducted based on the assumption of constipation being the etiology of PD; however, constipation and PD may mutually affect each other. 3) Further studies are necessary to explain the remaining 23.44% effect leading to PD by constipation.

## Introduction

Parkinson’s disease (PD) is a neurodegenerative disease manifested as both motor (such as tremor, bradykinesia, rigidity, and postural instability) and non-motor (including constipation, rapid eye movement sleep behavior disorder, and depression) symptoms (Rai et al., 2020; Rai and Singh, 2020; Rai et al., 2021). The worldwide prevalence was 0.3% in the general population with 40 years of age and older based on a meta-analysis of 47 studies (Pringsheim et al., 2014). An increasing trend of age-adjusted mortality in PD has been found (Rong et al., 2021). Risk factors for PD include age, sex, excess body weight, family history of PD, constipation, and so on (Stirpe et al., 2016). Constipation can be observed in PD patients as early as 20 years before the onset of motor symptoms (Savica et al., 2009). A recent retrospective cohort study based on the Taiwan National Health Insurance Research Database consisting of 551,324 participants free of PD found that the adjusted hazard ratio for developing PD was 3.28 (95% CI 2.14 to 5.03), 3.83 (95% CI 2.51 to 5.84), and 4.22 (95% CI 2.95 to 6.05) for individuals with different constipation severity categories (Lin et al., 2014). Another meta-analysis with a combined sample size of 741,593 participants found that those with constipation had a pooled odds ratio of 2.27 (95% CI 2.09 to 2.46) for developing subsequent PD when compared with those without constipation. In addition, constipation, which occurred more than 10 years prior to PD, had a pooled odds ratio of 2.13 (95% CI 1.78 to 2.56; *I* ^2^ = 0.0%) (Adams-Carr et al., 2016).

Although evidence has shown a strong correlation between constipation and PD, the detailed mechanism of constipation-related PD is still unclear. One of the possible pathogenic mechanisms could be through the alteration of the gut microbiome. Gut microbiota is a complex ecological community composed of trillions of microbes. It is able to influence both normal physiology and disease susceptibilities through bacterial metabolic activities and host interactions (Lozupone et al., 2012). At least 17 studies had reported dysbiosis of intestinal microbiota in PD patients (Keshavarzian et al., 2020). A 2-year follow-up study of 36 PD patients found that the counts of *Bifidobacterium*, *Bacteroides fragilis*, and *Clostridium leptium* were associated with PD severity (Minato et al., 2017). Several mechanisms of intestinal dysbiosis causing PD have been delineated in a recent review article, such as increased permeability of the intestinal barrier and the blood–brain barrier, increased inflammation and oxidative stress, changes in dopamine production, and molecular mimicry (Huang et al., 2021). Interestingly, intestinal dysbiosis can be developed in patients with chronic constipation (Ohkusa et al., 2019; Zhang et al., 2021), and this type of constipation-related intestinal dysbiosis could be cured by bisacodyl treatment, in which patients’ bowels were emptied by taking laxatives (Khalif et al., 2005). A mouse study has also shown that constipation was able to induce the dysbiosis of gut microbiota which further exacerbated experimental autoimmune encephalomyelitis (Lin et al., 2021). Although evidence of the association between constipation, gut dysbiosis, and PD has emerged, no studies have conducted an integral analysis to disentangle the role of intestinal microbial alteration in the mechanism between constipation and PD.

In this study, we hypothesized that constipation can cause PD *via* inducing intestinal dysbiosis. Mediation analysis was conducted to test and quantify the extent by which the intestinal microbial alteration explains the causal effect of constipation on PD incidence.

## Methods

### Participant Recruitment and Data Collection

We adapted our data from the study of Hill‐Burns et al. (Hill-Burns et al., 2017), in which 330 participants (185 men, 145 women, mean age 69.2) were enrolled from the NeuroGenetics Research Consortium during March 2014 to January 2015. The methods and the clinical and genetic characteristics of the NeuroGenetics Research Consortium dataset were described in detail in Hamza et al. (2010) Among the 330 participants, 199 (133 men, 66 women, mean age 68.4) were diagnosed with PD by the modified UK Brain Bank criteria. The remaining 131 controls (52 men, 79 women, mean age 70.4) were self-reporting free of neurodegenerative disease. Constipation symptoms were assessed by the Gut Microbiome Questionnaire, and six participants were excluded from this study due to no information about constipation status. The remaining 324 were included in our final data analysis. Details of the fecal sample collection process, DNA extraction and sequencing, and metadata collection can be found in Hill-Burns et al. (2017).

### Data Availability and Ethical Statement

Sequences analyzed in this study are accessible at the European Nucleotide Archive (ENA) under the accession number ERP016332. All data are open access and de-identified. No ethical approval is required.

### Processing of 16S rRNA Sequence Data

The 16S rRNA gene is highly conserved in bacteria. As a result, it is highly suited as a target gene for DNA sequencing for bacterial identification. The sequence reads were processed with Trimmomatic v0.39 (Bolger et al., 2014) to remove adaptors. The outputs were then processed, aligned, and categorized using DADA2 1.16 (Callahan et al., 2016). In brief, sequence reads were first filtered using DADA2’s recommended parameters. Filtered reads were then de-replicated and de-noised using DADA2 default parameters. After building the amplicon sequence variant (ASV) table and removing chimeras, taxonomy was assigned using SILVA v132 natively implemented in DADA2. We used the addSpecies function in DADA2 to add species-level annotation with SILVA as reference. Sequence counts were normalized to relative abundances (calculated by dividing the number of sequences that were assigned to a unique ASV by the total sequence count in the sample). Bacteria that exist in more than 10% of samples were used in later analysis. Regarding functional enrichment analysis, we used Phylogenetic Investigation of Communities by Reconstruction of Unobserved States (PICRUSt2) version 2.4.1 (Douglas et al., 2020) to infer metagenome composition in the samples, followed the recommended pipeline of normalizing ASVs by copy number (to account for differences in the number of copies of 16S rRNA between taxa), predicted functions using Kyoto Encyclopedia of Genes and Genomes (KEGG) (Kanehisa and Goto, 2000) orthologs, and grouped predicted pathways by KEGG hierarchical level 3.

### Statistical Analyses

We compared several demographic characteristics (including age, sex, race, BMI, residence location, alcohol consumption amount, smoking status, diet habit, and other neurological problems) between constipated and non-constipated groups by using the Wilcoxon test for continuous variables and the chi-square test for categorical variables. Analysis of the microbial difference between the two groups was performed using the Wilcoxon test. We also compared the overall taxonomic diversity between groups by calculating alpha and beta diversity values that incorporate both species richness and evenness. Regarding alpha diversity, we estimated the observed richness (i.e., number of ASVs) and the Chao1, Shannon, and Simpson indices from the ASV table (Chao, 1984; Magurran, 1988; Rosenzweig, 1995) using phyloseq 1.32.0 (McMurdie and Holmes, 2013). *p*-values for alpha diversity were calculated with ANOVA using stats 4.0.5. Regarding beta diversity, we estimated the dissimilarities (distance) between the two groups using the following metrics to ensure that the choice of metrics did not affect the results: unweighted unique fraction metrics (UniFrac), weighted UniFrac (Lozupone et al., 2011), and Canberra distance (Lance and Williams, 1967). Beta diversity indices for weighted and unweighted UniFrac were calculated with phyloseq 1.32.0 (McMurdie and Holmes, 2013). Canberra distance was calculated with vegan 2.5.7. *p*-values for beta diversity were calculated with ADONIS using vegan 2.5.7.

The mechanism of constipation causing PD is assumedly mediated by microbiome alterations. Mediation analysis was conducted for quantifying the extent by which the intestinal microbial alteration explained the causal effect of constipation on PD. Constipation status is the exposure variable, PD status is the outcome of interest, and intestinal microbial alteration is the mediator. Sex, age, and the amount of fruits and vegetables taken were adjusted for the following mediation analysis. The details of conducting the mediation analysis are shown in the **Appendix**. All statistical analyses were performed with R version 3.6.0.

## Results

We compared the general characteristics between the constipation (*N* = 31) and non-constipation (*N* = 293) groups and the results are summarized in **Table 1**. There were no significant differences in age, sex, race, height, weight, BMI, geographic area (latitude, longitude, location), alcohol and coffee consumption amount, smoking, diet habit (eat fruits or vegetables daily, eat grains daily, eat meat daily, eat nuts daily, eat yogurt daily), and the presence of neurological problem except PD. The presence of PD was significantly higher in the constipated group (93.5% vs. 57% in the non-constipated group; *p*-value < 0.001).

**Table 1:** Comparing patients with and without constipation on demographic variables.

	No (*N* = 293)	Yes (*N* = 31)	*p*-value
Age			
Mean (SD)	69.2 (8.77)	69.6 (9.86)	0.992
Sex			
Female	133 (45.4%)	9 (29.0%)	0.120
Male	160 (54.6%)	22 (71.0%)	
Race			
White	290 (99.0%)	30 (96.8%)	0.354
More than one race	2 (0.7%)	1 (3.2%)	
Black or African American	1 (0.3%)	0 (0%)	
Height (in.)			
Mean (SD)	67.2 (3.95)	68.6 (3.95)	0.101
Missing	3 (1.0%)	0 (0%)	
Weight (lbs)			
Mean (SD)	174 (40.1)	187 (35.7)	0.062
Missing	7 (2.4%)	2 (6.5%)	
BMI			
Mean (SD)	27.0 (5.52)	28.1 (4.92)	0.193
Missing	7 (2.4%)	2 (6.5%)	
Latitude			
Mean (SD)	−33.0 (81.1)	−43.1 (83.2)	0.751
Location			
Atlanta, GA	42 (14.3%)	2 (6.5%)	0.459
Albany, NY	119 (40.6%)	13 (41.9%)	
Seattle, WA	132 (45.1%)	16 (51.6%)	
Longitude			
Mean (SD)	−20.6 (61.9)	−11.8 (62.4)	0.319
Alcohol amount			
Mean (SD)	0.966 (0.831)	0.933 (0.785)	0.853
Missing	3 (1.0%)	1 (3.2%)	
Smoke			
No	275 (93.9%)	28 (90.3%)	0.591
Yes	16 (5.5%)	3 (9.7%)	
Missing	2 (0.7%)	0 (0%)	
Coffee amount			
Mean (SD)	1.22 (0.848)	1.30 (0.794)	0.670
Missing	4 (1.4%)	1 (3.2%)	
Eats fruits or vegetables daily			
No	48 (16.4%)	9 (29.0%)	0.141
Yes	241 (82.3%)	22 (71.0%)	
Missing	4 (1.4%)	0 (0%)	
Eats grains daily			
No	90 (30.7%)	10 (32.3%)	0.998
Yes	196 (66.9%)	20 (64.5%)	
Missing	7 (2.4%)	1 (3.2%)	
Eats meats daily			
No	117 (39.9%)	13 (41.9%)	1
Yes	171 (58.4%)	18 (58.1%)	
Missing	5 (1.7%)	0 (0%)	
Eats nuts daily			
No	215 (73.4%)	25 (80.6%)	0.606
Yes	73 (24.9%)	6 (19.4%)	
Missing	5 (1.7%)	0 (0%)	
Eats yogurt daily			
No	259 (88.4%)	28 (90.3%)	1
Yes	24 (8.2%)	3 (9.7%)	
Missing	10 (3.4%)	0 (0%)	
Other neuro problems			
No	263 (89.8%)	28 (90.3%)	0.494
Yes	13 (4.4%)	0 (0%)	
Missing	17 (5.8%)	3 (9.7%)	
Parkinson’s disease			
No	126 (43.0%)	2 (6.5%)	<0.001
Yes	167 (57.0%)	29 (93.5%)	

**Figure 1:**
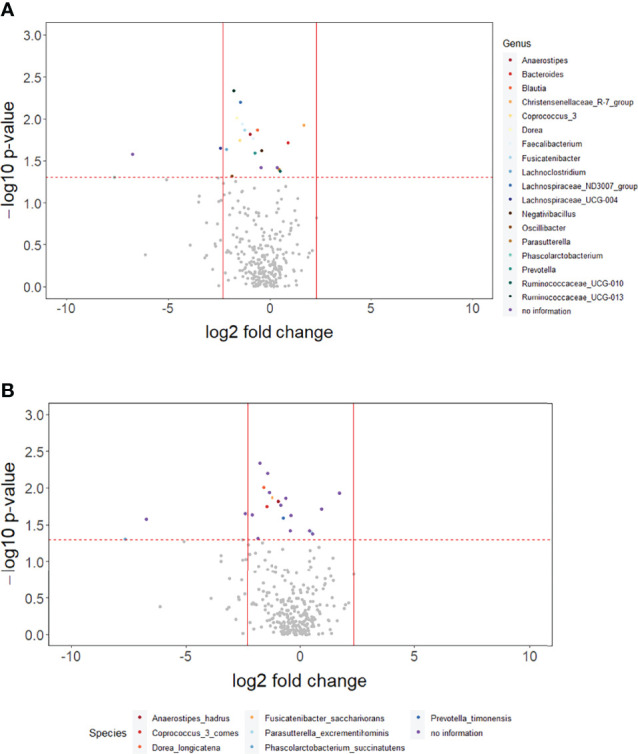
The overall difference of gut microbiota between the constipated group and the non-constipated group was assessed. A negligible number 2*10^-6^ was added to the abundance of every amplicon sequence variant (ASV) to avoid the value of the magnitude of fold change being infinitely large or small. This number was generated based on the 100th of the minimal relative abundance. Each point represents an ASV with its magnitude fold change in relative abundance (log2 of the constipated group/non-constipated group) on the *x*-axis and the value of statistical significance (−log10 of *p*-value) on the *y*-axis. The dashed red line shows where *p* = 0.05 with points above the line having *p <*0.05 and points below the line having *p >*0.05. Significant ASVs are colored based on genus **(A)** and species **(B)**. Points outside of the solid lines are ASVs with a mean abundance of 0 in either the non-constipation group (left) or the constipation (right) group.

**Figure 2:**
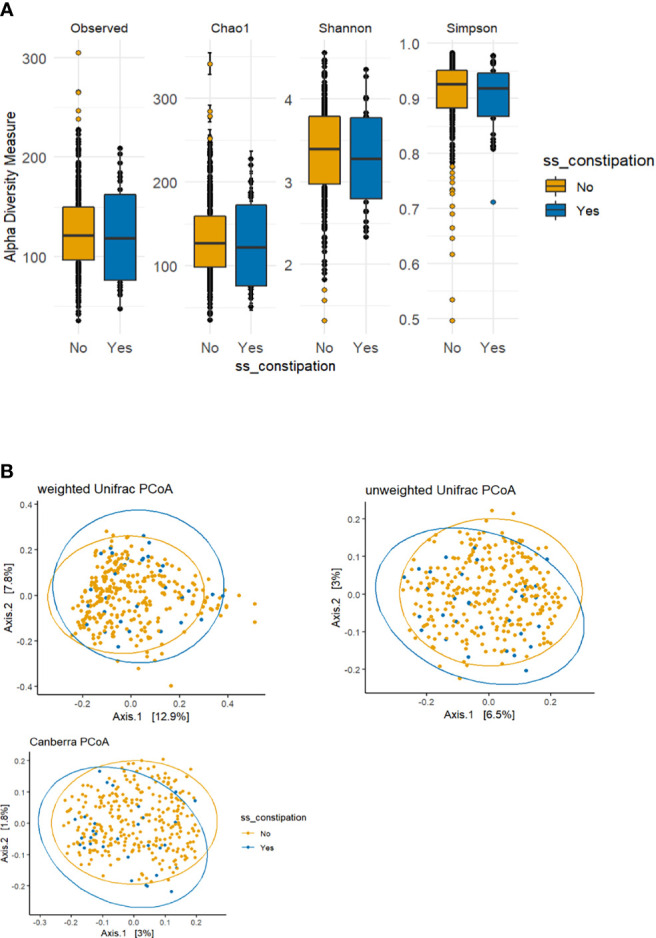
Alpha and beta diversity plots to visualize the difference in microbiota structure between the constipated group versus the non-constipated group. **(A)** The boxplot figures show the alpha diversity of the bacterial communities by means of observed amplicon sequence variants (ASVs) and Chao1, Shannon, and Simpson indices. Median and lower and upper quartiles are shown on the plots. ss_constipation, status of constipation. **(B)** The principal component analysis (PCoA) plots with the following three distance measures: Canberra, unweighted unique fraction metric (UniFrac), and weighted UniFrac.

The *p*-values for the comparison of microbial differences in relative abundance between the constipated group and the non-constipated group are shown in **Figure 1**, under the genus (**Figure 1A**) and species levels (**Figure 1B**). Eighteen genera and seven species were significantly different between the groups. The results of overall taxonomic alpha and beta diversity between the groups are shown in **Figure 2**. Regarding alpha diversity, none of the four metrics (observed, Chao1 index, Shannon index, Simpson index) were significantly different between the groups (*p*_Observed_ = 0.6258; *p*_Chao1_ = 0.5654; *p*_Shannon_ = 0.5335; *p*_Simpson_ = 0.7536) (**Figure 2A**). In contrast, all three metrics of beta diversity (unweighted UniFrac, weighted UniFrac, Canberra distance) showed significant changes in the community structure between the two groups (*p*_Unweighted-unifrac_ = 0.0001; *p*_Weighted-unifrac_ = 0.0003; *p*_Canberra_ = 0.0001) (**Figure 2B**).

In order to understand the key microbial alterations that mediated constipation-related PD, mediation analysis was performed, in which the results are shown in **Table 2**. The proportion mediated by intestinal microbial alteration was 76.56% at the genus level (*p* = 0.0177) (**Table 2A**), 69.8% at the species level (*p* = 0.0309) (**Table 2B**), and 66.27% in functional pathways (*p* = 0.0445) (**Table 2C**), respectively. Nine genera had a significant mediation effect, which were *Anaerostipes* (*p* = 0.0043), *Fusicatenibacter* (*p* = 0.0048), *Lachnospiraceae_ND3007_group* (*p* = 0.0076), *Blautia* (*p* = 0.0077), *Ruminococcaceae_UCG_013* (*p* = 0.0097), *Coprococcus_3* (*p* = 0.0155), *Faecalibacterium* (*p* = 0.0293), *Dorea* (*p* = 0.0322), and *Lachnospiraceae_UCG_004* (*p* = 0.033). Four species had a significant mediation effect, namely, *Anaerostipes_hadrus* (*p* = 0.0043), *Fusicatenibacter_saccharivorans* (*p* = 0.0048), *Coprococcus_3_comes* (*p* = 0.0155), and *Dorea_longicatena* (*p* = 0.0482). The aforementioned bacteria were all contained in the significant taxa list presented in **Figure 1**. Lipid acid metabolism was the only pathway that had been found to have a significant mediation effect.

**Table 2:** Mediation analysis for the mechanism of constipation inducing PD mediated by intestinal microbial alteration with different measures as mediators: (A) genus level, (B) species level, and (C) functional pathway level.

	Estimate (SE)	PM (%)	*p*-value
(A)			
Unadjusted	2.3597 (0.7555)		
Adjusted for			
All genera	0.5530 (0.0977)	76.56%	
* Anaerostipes*	2.1552 (0.7549)	8.66%	0.0043
* Fusicatenibacter*	2.1843 (0.7596)	7.43%	0.0048
* Lachnospiraceae_ND3007_group*	2.1513 (0.7594)	8.83%	0.0076
* Blautia*	2.3521 (0.7710)	0.32%	0.0077
* Ruminococcaceae_UCG_013*	2.1835 (0.7576)	7.47%	0.0097
* Coprococcus_3*	2.2151 (0.7569)	6.13%	0.0155
* Faecalibacterium*	2.2783 (0.7581)	3.45%	0.0293
* Dorea*	2.2375 (0.7545)	5.17%	0.0322
* Lachnospiraceae_UCG_004*	2.2338 (0.7590)	5.33%	0.0330
(B)			
Unadjusted	2.3597 (0.7555)		
Adjusted for			
All species	0.7127 (0.1064)	69.8%	
* Anaerostipes_hadrus*	2.1552 (0.7549)	8.66%	0.0043
* Fusicatenibacter_saccharivorans*	2.1843 (0.7596)	7.43%	0.0048
* Coprococcus_3_comes*	2.2151 (0.7569)	6.13%	0.0155
* Dorea_longicatena*	2.2280 (0.7566)	5.58%	0.0482
(C)			
Unadjusted	2.3597 (0.7555)		
Adjusted for			
All functional pathways	0.7960 (0.1865)	66.27%	
I_Lipoic_acid_metabolism	2.2630 (0.7621)	4.09%	0.0276

PM, proportion mediated; SE, standard error; CI, confidence interval; I_Lipoic_acid_metabolism, metabolism of cofactors and vitamins/lipoic acid metabolism.

## Discussion

Since Braak first proposed the gut–brain axis hypothesis in 2003 (Braak et al., 2003; Braak et al., 2003), the GI system had been hypothesized as the origin of Parkinson’s disease. Through a series of elegant neuropathological studies in postmortem PD patients, Braak and his colleagues proposed a hypothesis in which toxins or pathogens enter the host through the GI tract, causing inflammation and aggregation of alpha-synuclein protein in the enteric nervous system; this aggregated alpha-synuclein protein moves up to the central nervous system *via* the vagus nerve, resulting in the degeneration of dopaminergic neurons in the substantia nigra (Hawkes et al., 2007; Keshavarzian et al., 2020). This hypothesis also explains why PD patients develop gastrointestinal symptoms, such as constipation, prior to the onset of the cardinal motor and central nervous system symptoms. Despite the evidence of constipation-related PD being microbially associated, no studies have provided direct evidence of their causal relationship. To the best of our knowledge, this is the first study to examine the mechanism of constipation-causing PD mediated by microbiota by using mediation analysis. We also found 18 genera and 7 species and beta diversity which were significantly different between the constipated and non-constipated groups, indicating that constipation was associated with intestinal dysbiosis and was corresponding to the findings in previous studies (Khalif et al., 2005; Ohkusa et al., 2019; Zhang et al., 2021). It was estimated that 76.56% of the effect of constipation-related PD was mediated by microbiota at the genus level, providing evidence that strengthened the causal effect of constipation on PD. Our results indicate that the benefit of probiotics prescription probably prevents around 76.56% of the incidence of constipation-related PD.

Bacteria significantly related to the mediation mechanism in this study had all been found strongly related to PD in previous studies (Hill-Burns et al., 2017; Li et al., 2017; Petrov et al., 2017; Lin et al., 2018; Aho et al., 2019; Li et al., 2019; Romano et al., 2021). They were also a subset of the constipated-related bacteria shown in **Figure 1**.

Based on the results of a correlation network analysis in a previous study, PD-associated bacterial genera can be mapped to three polymicrobial clusters: opportunistic pathogens, short-chain fatty acid (SCFA-producing bacteria, and carbohydrate-metabolizing probiotics (Wallen et al., 2020). The bacteria found in our study with significant mediation effect of constipation on PD all fall into the SCFA-producing category (*Anaerostipes*, *Fusicatenibacter*, *Coprococcus*, *Dorea*, *Blautia*, *Faecalibacterium*, *Ruminococcaceae_UCG_013*, *Lachnospiraceae_ND3007_group*, and *Lachnospiraceae_UCG_004*). SCFAs, including acetate, propionate, and butyrate, are the major products of microbial fermentative activity in the gut. A low level of SCFAs, especially butyrate, had been associated with increased intestinal permeability, resulting in a leaky gut (Dalile et al., 2019). SCFAs also enhance the integrity of the blood–brain barrier through regulating the maturation of microglia by enabling the microglial expression of SCFA-responsive genes such as histone deacetylase (Dalile et al., 2019). Reduced levels of SCFA-producing bacteria in PD patients had been confirmed in many studies and also in other inflammatory diseases such as IBD, alcohol-associated pathology, and metabolic syndrome (Koh et al., 2016). Our study demonstrated that constipation can cause PD through reducing SCFA-producing bacteria, which further increase intestinal permeability, lead to endotoxin or exotoxin penetration, and induce subsequent pathological change in PD.

*Akkermansia*, *Lactobacillus*, and *Bifidobacterium* are anti-inflammatory, carbohydrate-metabolizing probiotics, which had been found increasing in PD patients (Hill-Burns et al., 2017; Petrov et al., 2017; Aho et al., 2019; Barichella et al., 2019; Romano et al., 2021). However, we did not find a significant mediation effect on these bacteria, which may indicate that increased carbohydrate-metabolizing probiotics in PD patients did not have a causal role in the pathogenesis of PD but more likely to be a compensatory change to overcome intestinal dysbiosis.

Alpha-lipoic acid (ALA) is a naturally occurring enzyme cofactor with antioxidant properties and has known neuroprotective effects on PD (Spalding and Prigge, 2010). An animal study showed that ALA can decrease intracellular levels of reactive oxygen species, promote the survival of dopaminergic neurons, and improve motor deficits of a PD animal model (Tai et al., 2020). Our study found a significant mediation effect of lipoic acid metabolism, indicating that lipoic acid metabolism may have a causal role in the pathogenesis of PD.

Three limitations were worthy to note in this study. First, only when all potential baseline confounders were adjusted could causal effects be unbiasedly estimated by regression coefficients. However, several potential confounders, such as the frequency of regular physical activities (Mika et al., 2015; Monda et al., 2017) and medicinal plant intake (e.g., *Mucuna pruriens*) which may have roles in relieving constipation that could further ameliorate intestinal dysbiosis, were not provided in the original dataset. Second, constipation, intestinal microbial alteration, and PD may mutually affect each other. Our study was conducted based on the assumption of constipation being the etiology of PD, but PD could also reversely accelerate the severity of constipation. This similar bidirectional causal relation applies to PD and microbial alteration, in which alpha-synuclein protein that aggregates in the central nervous system could move down to the intestinal system and further induce microbial alteration. This microbial alteration could also lead to constipation. The bidirectional causality stated above is termed “time-varying” issues in the literature (Lin et al., 2017; VanderWeele and Tchetgen Tchetgen, 2017; Hernán and Robins, 2018). A sophisticated model should be adapted if microbiome and constipation status are measured repeatedly in longitudinal follow-up studies. For those types of studies, PD medications such as entacapone which potentially lead to constipation (Fu et al., 2022) should be considered a candidate mediator or time-varying confounder. A comparison of the microbial alteration between the constipation versus the non-constipation groups among PD patients would also be necessary. Corresponding animal studies by providing probiotics to validate the aforementioned mechanisms are required as well. Finally, our results explain 76.56% of the mechanism of constipation-related PD, while the remaining 23.44% is still unclear. Exotoxin aggregation could possibly be involved in the unknown mechanism. Further studies are necessary to confirm the remaining factors leading to PD by constipation.

## Conclusion

Our findings support that gut dysbiosis plays a critical role in the pathogenesis of constipation-related PD, mostly through the decreasing of SCFA-producing bacteria, indicating that probiotics with SCFA-producing bacteria may be promising in the prevention and treatment of constipation-related PD.

## Data Availability Statement

The original contributions presented in the study are included in the article/**Supplementary Material**. Further inquiries can be directed to the corresponding authors.

## Author Contributions

S-CF came up with the original idea. S-CF, Y-CH, and P-HW set up and performed the bioinformatics procedures. C-HL, Y-CH, and P-HW conducted the data analysis. L-CS wrote the first version of the manuscript. S-CF, S-HL, and HW contributed to the paper. All authors approved the final version of this article.

## Funding

This research was supported by the Ministry of Science and Technology in Taiwan (107-2118-M-009-002-MY2 and 110-2636-B-009-001-MY4).

## Conflict of Interest

The authors declare that the research was conducted in the absence of any commercial or financial relationships that could be construed as a potential conflict of interest.

## Publisher’s Note

All claims expressed in this article are solely those of the authors and do not necessarily represent those of their affiliated organizations, or those of the publisher, the editors and the reviewers. Any product that may be evaluated in this article, or claim that may be made by its manufacturer, is not guaranteed or endorsed by the publisher.
