# Supplementary material for: Exploring the Causal Effect of Constipation on Parkinson’s Disease Through Mediation Analysis of Microbial Data
Source: Front Cell Infect Microbiol. 2022 May 11;12:871710. doi: 10.3389/fcimb.2022.871710 (PMC9130588; doi:10.3389/fcimb.2022.871710)
Supplement: Supplementary file 1 [file DataSheet_1.pdf]

Appendix of "Exploring the Causal Effect of Constipation on Parkinson's Disease through Mediation Analysis of Microbial Data"

Shih-Chen Fu[1], Ling-Chieh Shih[2], Pei-Hua Wu[1], Yi-Chen Hsieh[1], Chung-Han Lee[1], Sheng-Hsuan Lin[1,*], Hsiuying Wang[1,]**

[1] Institute of Statistics, National Yang Ming Chiao Tung University, Hsinchu

[2] Department of Medicine, National Yang Ming Chiao Tung University, Taipei, Taiwan

[*] shenglin@stat.nctu.edu.tw

[**] wang@stat.nycu.edu.tw

Details of mediation analysis

For conducting mediation analysis, we constructed three statistical models. In Model 1, we built a regular logistic regression with PD as the dependent variable while constipation and baseline confounders as independent variables. In Model 2, we built another logistic regression with PD as the dependent variable while constipation, gut microbial alteration, and baseline confounders as independent variables. Because microbial alteration is a high dimensional variable, we adapted a ridge regression algorithm. In Model 3, we built a linear regression model with gut microbial alteration as a dependent variable while constipation and baseline confounders as independent variables. Here the coefficient of constipation in Models 1 and 2 was interpreted as the effect of constipation on PD and the direct effect (the effect of constipation on PD that is not mediated through intestinal microbial alteration), respectively. The coefficient of constipation in Model 3 represented the effect of constipation on microbial alteration, and the coefficient of gut microbial alteration in Model 3 represented the effect of each measurement of intestinal microbial alteration on PD. The mediation effect (the effect of constipation on PD that is mediated through any intestinal microbial alteration) was measured by the difference of the coefficients of constipation between Model 1 and 2. Joint hypothesis tests were used for calculating p-values of the mediation effect. All statistical analyses were performed with R version 3.6.0., under which Ridge regression analysis was performed using glmnet (version 4.1-2) and linear model and logistic regression were built and performed using stats (version 4.1.0).
